# Comparing in-person and remote consent of people with dementia into a primary care-based cluster randomised controlled trial: lessons from the Dementia PersonAlised Care Team (D-PACT) feasibility study

**DOI:** 10.1186/s12874-025-02685-0

**Published:** 2025-10-30

**Authors:** T. M. Oh, S. Batool, C. Musicha, L. Greene, H. Wheat, L. Smith, S. Griffiths, A. Gude, L. Weston, H. Shafi, K. Stevens, C. Sutcliffe, W. Taylor, W. Ingram, B. Hussain, P. Clarkson, I. Sherriff, O. C. Ukoumunne, S. Creanor, R. Byng

**Affiliations:** 1 Community and Primary Care Research Centre, University of Plymouth, Plymouth, UK; 2 Social Care and Society, University of Manchester, Manchester, UK; 3 Medical Statistics, University of Plymouth, Plymouth, UK; 4 Peninsula Clinical Trials Unit, University of Plymouth, Plymouth, UK; 5 NIHR Applied Research Collaboration South West Peninsula (PenARC), University of Exeter, Exeter, UK; 6 Exeter Clinical Trials Unit, University of Exeter Medical School, University of Exeter, Exeter, UK; 7 Centre for Ageing Population Studies, University College London, London, UK; 8 Exploristics Ltd., Belfast, UK; 9 Focused Care CIC, Oldham, UK; 10 University of Nottingham, Nottingham, UK; 11 Liverpool John Moores University, Liverpool, UK

**Keywords:** Dementia, Primary care, Clinical trial, Capacity, Consent, Recruitment challenges, Remote consent, In-person consent, COVID-19, Dementia PersonAlised Care Team (D-PACT)

## Abstract

**Background:**

Complex socio-cultural, psychological, geographical, and service-related challenges are faced when recruiting people with dementia for clinical trials. The aim of Phase 1 of the Dementia PersonAlised Care Team (D-PACT) project was to assess the feasibility of recruiting (identifying, approaching and consenting) people with dementia, including those without capacity to consent, to a cluster randomized controlled trial of a primary care-based personalized dementia support intervention in England. COVID-19 necessitated a shift to remote working, creating the opportunity to compare recruitment strategies before and under lockdown constraints. This paper shares the adaptations made to enable remote consent and capacity judgement with people with dementia, as well as lessons learned.

**Methods:**

Consent was conducted in person from September 2019 to March 2020. Remote consent was implemented from September 2020 to March 2021 after an enforced pause. Both quantitative and qualitative data were collected. Recruitment rates (proportion consented from eligible patients approached), mean monthly consent rates, and time spent on consent-related activities (tasks before and after consent/capacity-judgment meetings, miscellaneous tasks, travel) were compared. Participant experiences with remote recruitment were examined through thematic analysis of qualitative interviews.

**Results:**

Pre-COVID-19, 22 participants (9.9%) out of 228 approached were consented in person. During the pandemic, 19 participants (9.6%) out of 198 were consented remotely, excluding 15 participants initially approached pre-pandemic and later consented via remote means. Mean monthly consent rates increased from 3.6 (in person) to 5.6 (remote). However, remote consent required more time (mean 14 researcher-hours per participant vs. 9 in person), with 75% of time spent on consent-related tasks compared to 20% in person. Travel accounted for 40% of in-person consent time. Interviews (n = 13) showed general acceptability of remote processes. However pre-consent information was perceived as excessive and led some participants to skim materials, potentially reducing understanding.

**Conclusions:**

While remote consent is time-intensive, it achieves comparable rates (proportion consented/total approached) to in-person methods and higher monthly consent rates. A flexible, hybrid approach can enhance participation, offer choice, and increase person-centredness. Realistic planning for time and resources is crucial for inclusive dementia research. Funders should support these needs to ensure effective recruitment.

**Trial registration:**

ISRCTN80204146.

## Background

Recruiting people with dementia for research is challenging [1]. Reasons include participant-related factors e.g., unwillingness or inability to participate [2], health status, family context [3], communication needs, fluctuating cognition, or lack of capacity. Barriers introduced (often inadvertently) by the research team [4] or associated with the nature of the research also contribute to low uptake, particularly in studies of complex interventions like physical exercise trials [2]. Additional challenges arise in primary care recruitment, with over two-thirds of health studies not reaching their patient targets [5, 6]. These difficulties are exacerbated by the low participation rate among General Practice (GP) surgeries – in 2019/20 only 36% of GP practices in the UK were research active [7].

COVID-19 restrictions presented clinical trials, including ours, with unprecedented challenges including how to recruit (i.e. identify, approach and consent) new participants with social distancing and lockdowns in place [8]. Our project – the Dementia PersonAlised Care Team (D-PACT) – was a five-year research programme funded by the National Institute for Health and Care Research (NIHR) to develop and evaluate a personalised support intervention involving a trained dementia support worker (DSW) in primary care, aimed at people with moderate to severe dementia (some of whom may have lost capacity to consent) and their carers [9]. During Phase 1 (Feasibility) we developed and tested the feasibility of our recruitment process, designed to align with person-centred principles of dementia care and be more suited to the needs of people with dementia; this is described elsewhere [10]. Individuals lacking mental capacity are under-represented in research [11–13] and challenges specific to these individuals were considered in our materials and design of the recruitment process [10].

Recruitment to the feasibility study was in progress when the first UK-wide COVID-19 lockdown began in March 2020. All trials were paused while research teams waited for Government guidance. Pre-pandemic, our approach process combined in-person contact (generally assumed until then to be preferred and easier for people with dementia) with other forms of contact, e.g. letters and phone calls, with the main in-person contact happening during the consent meeting. This meeting, which comprised judging a potential participant’s capacity to consent as well as the consent process (via consultee for those lacking capacity to consent) was conducted in person at the person’s home or at their GP surgery premises. To enable us to continue recruitment throughout the lockdowns we adapted the consent process so that it could be conducted remotely. We were mindful that increased communication needs among some people with dementia and the impact of isolation on mood and cognitive functioning [14] might need to be considered.

We were already aware of published papers on barriers to – and the ethics of – including people with dementia (and who lack capacity) in research [11, 13, 15–17] via our earlier work to develop our recruitment strategy for Phase 1 (pre-pandemic). In order to make the necessary adaptations to suit remote working we searched for any available guidance and literature relevant to recruiting participants *remotely* and identified a few papers describing a range of strategies for remote recruitment [18–21]. At least two studies found that comprehension in informed consent obtained via telemedicine was comparable to that of in-person informed consent [22, 23] although many factors, including medium of communication, do need to be considered to ensure adequate understanding has been achieved [24, 25]. A handful of studies described using digital tools to gain informed consent in paediatric populations [26] and from people with schizophrenia, who may have impaired decision-making ability compared to healthy individuals [27, 28]. However we found nothing published on how to remotely judge capacity *and* gain consent from people with dementia.

Three principles emerged from a review of (and team discussions about) the identified literature above. The first principle was to ensure that any adaptations remained person-centred, inclusive, and dementia-friendly. This was driven by our commitment to including people [15] who might otherwise be excluded from research because of complex legal frameworks factors and what Shepherd [13] calls a “paternalistic” stance in the protection of people lacking capacity from perceived harms that may arise from research. The second principle was to maintain participant protection. Finally, maintaining researcher well-being was prioritized.

This paper aims to: (i) describe the process we put into place to remotely consent and judge the capacity of people with dementia to a primary care-based study (see Adaptations to enable remote consent during COVID-19); (ii) show, albeit based on a limited amount of feasibility data, how recruitment during the pandemic using remote consent compared to recruitment pre-pandemic, where consent took place in person; and (iii) report how a subset of participants viewed the remote consent process. The potential of a hybrid model of recruitment is proposed, where potential participants are provided the choice of being consented in-person or remotely. Our study adds to the small number of recent studies reporting their own adaptations to allow remote working [29, 30] and fills a gap by showing *how* to do this when including people who may lack capacity in research studies.

## Methods

### Study design

Phase 1 of D-PACT explored the feasibility of a cluster randomised controlled trial (cRCT) to evaluate a DSW role based in primary care. A favourable ethical opinion from the South Central – Berkshire NHS Research Ethics Committee (reference: 19/SC/0264) was received (including for any amendments), and the study was registered with the ISRCTN registry (registration number: ISRCTN80204146).

We applied a mixed-methods approach in Phase 1, collecting both quantitative and qualitative data. However we focus here only on those data relevant to the aims of this paper. In order to examine the impact of adapting our in-person consent of people with dementia to remote consent, we examined data from the two active recruitment phases, i.e. September 2019 – March 2020 (in-person consent, pre-COVID-19) and September 2020 – March 2021 (remote consent, during COVID-19).

### Recruitment target

As this was a feasibility study of a cRCT, allocation occurred at the level of the GP practices. GP practices in Southwest (SW) (*n* = 4) and Northwest (NW) (*n* = 4) England were recruited and allocated to receive the intervention or treatment as usual (TAU). In each region, the first recruited practice was allocated to the intervention group to maximise learning about the role of the DSW within primary care and to inform the recruitment process in an iterative fashion. The remaining three practices in each region were randomly allocated to intervention or TAU at a ratio of 2:1 to allow a higher number of participants to receive the intervention so that adequate material would be collected for the parallel formative evaluation. In each region, the participant recruitment target for the first non-randomised practice was 10 people with dementia (and carers if available/willing) and then 15 for each of the remaining three practices (i.e. recruitment target total = 55 people with dementia per region). Except for the first two non-randomised GP practices, the recruiting team were blind to the allocation of the remaining practices. Changes to the number of participating GP practices occurred because of the pandemic (see Results, Proportion recruited from those approached).

### Adaptations to enable remote consent during COVID-19

The pre-pandemic recruitment strategy (i.e. identification, approach and consent) we developed has been reported elsewhere [10]. A comparison of the pre-pandemic and pandemic “identification” and “approach” components of the recruitment strategy can be found in Appendix A. Here we will focus on the “consent” component of the recruitment strategy, describing how we adapted the in-person process to suit the restrictions imposed by COVID-19 on in-person contact (see also Table 1 for an overview).

**Table 1:** Comparison of the consent meeting before and during COVID-19

Recruitment component	In-person (pre-COVID-19)	Remote (during COVID-19)
Consent and capacity judgement Consent meeting (“study involvement meeting”), including assessment of capacity to consent	Research team sent materials to potential participants before the consent meeting: - Information about the study sent (short PIS or full PIS if appropriate, and brief study information) Consent meeting at the person’s home or GP surgery; researcher brought other supporting documents and baseline questionnaires in case consented participant wanted to continue	Research team sent materials to potential participants before the consent meeting: - Information about the study sent (full PIS and brief study information) - Supporting documents (communication tool; information on Zoom, etc.) - Baseline questionnaires Pre-consent meeting technical check (remote) by a researcher Consent meeting over video or telephone call with a researcher

#### Pre-pandemic consent meeting

In our study eligible patients in participating GP practices were identified and approached using a multi-stage strategy [10] (see also Appendix A). Patients who were interested to learn more returned a positive “expression of interest” (EoI) form, providing consent to be contacted by researchers to organise the consent (and capacity judgement) meeting. Prior to COVID-19 the consent meeting happened at the person’s home or at their GP surgery. At this meeting researchers provided a full description of study aims, duration of participation, trial processes (including randomisation) and content (e.g. the intervention). Originally there were two full-length participant information sheets (PIS), one for the person with dementia and one for the carer. Later a shortened PIS for the person with dementia was posted prior to the meeting, with researchers going through the full PIS (one for the person with dementia, one for the carer) at the study involvement meeting itself [10]. However this was later amended because of the burden of going through a lot of information at the visit, and so we offered the person with dementia and their carer/family the option to receive the full PIS in advance of the meeting to provide them the chance to read the information in their own time and pace and prepare questions to ask at the meeting. Once the researcher had gone through the PIS with the person with dementia and carer at the visit, the consent process would commence if they wanted to go forward and did not want more time to decide. Researchers would formally assess the person’s capacity to consent as part of this (“capacity judgement”, described in detail elsewhere [10]) and introduce the option for a consultee declaration if needed. In accordance with the Mental Capacity Act [31], potential participants were assumed to have capacity unless there was good reason to believe otherwise. In the latter case, the option of a consultee declaration was offered although this needed to be done sensitively to avoid any sense of having failed an imagined test. Researchers also paid attention to signs of assent or dissent in the case where someone demonstrated lack of capacity to consent; verbal or non-verbal cues from the person with dementia were noted. As described previously [10] our procedure was designed to allow the potential participant with dementia several opportunities to have main elements of the study explained, ask questions and describe in their own words what they understood the research to be about and what their participation involved. If needed, a communication tool [10] we developed to aid this process was used. This tool also supported individuals who might be non-verbal or have limited verbal output to demonstrate capacity. Two researchers attended the in-person study involvement meeting, guided by recommended wording for explanations and questions prepared by the research team. The team agreed that the nature of these meetings should be unhurried (up to 2 h) to enable a truly person-centred approach. Prior to lockdowns, participants who had completed the consent process were then administered the Addenbrooke’s Cognitive Examination-III [32] (ACE-III) to check final eligibility for the trial before proceeding. This was carried out after consent to enable us to include these data in our analyses. Although researchers offered to return to collect baseline data, consented participants usually preferred for the researchers to do this at the same meeting (researchers did use their judgement as well, noting if the participant was fatigued and taking a rest or returning at an agreed time). One researcher conducted this with the person with dementia (if comfortable being on their own) and the second researcher with the carer. Questions were read out to participants, who could read their copy if they chose.

#### Consent meeting during the pandemic

The consent meeting was adapted so it could take place remotely either over a telephone or video call during COVID-19. Likewise, we adapted materials as necessary. Initial adaptations were informed by our own team’s expertise in person-centred recruitment [10, 15, 33] and the three principles described in the Introduction above. Feedback from our peer research group (D-PACT patient and public involvement group) led to further refinement, and researchers conducted dry runs over Zoom before recruitment during the pandemic period commenced.

Although we had already made considerable efforts to co-design participant-friendly materials for when we began (in-person) recruitment, we reviewed these to ensure that they could work in a remote meeting. For example, the communication tool [10] was amended to include instructions to the person with dementia or carer on how to use it during the remote meeting. Before the pandemic these extra documents would be brought to the meeting by the researcher; during the pandemic it was necessary to post them prior to the meeting so the potential participant could have the documents on hand. New documents to support remote communication were also included in the pre-meeting package, e.g. Skype/Zoom instructions. In addition to the PIS and materials to support consent, a copy of the questionnaires that would be used during the baseline data collection (whether directly after consent or at a separate telephone or video call) was sent. These were sent at the same time to reduce the number of times researchers left home while lockdowns were in place. This had the impact of increasing material received by the potential participant. Another change was to obtain verbal, rather than written, consent because we did not want to add extra risk to the dyad during the pandemic by requiring a signed consent form to be posted (second principle to maintain participant protection). A researcher would thus audio record going through the consent form with the person with dementia or consultee form with the carer, including the providing of consent or consultee’s judgement that the person with dementia would have wanted to participate. Researchers confirmed on their copy of the consent form that consent had been verbally recorded and recordings were stored in a secure location on the University’s servers. One other change was the reduction of two researchers to one. Having two as we did pre-pandemic was particularly difficult if the dyad had chosen the telephone as the mode of communication – during lockdown researchers were also confined to their own homes and did not have conference calling capabilities (i.e. participants in more than two locations on a call) on their telephone. If video call was the mode of contact, we felt that having two researchers on screen might add to the “noise” and place higher burden on the person with dementia (especially in those early days of using video calls before many people became familiar with using them).

Final eligibility in terms of cognitive status was still required to be confirmed post-consent. During the pandemic, the Modified Telephone Interview for Cognitive Status [34] (TICS-m, designed to assess cognitive function over the telephone) replaced the ACE-III, which is only validated to be captured face-to-face. Every effort was made to assign the same researcher to a potential participant, and researchers used any opportunity to start to build a picture of the person with dementia’s cognitive status (e.g. pre-consent telephone calls).

Researchers received training (including opportunities to practice) and regular peer support or supervision; debriefs following consent meetings were also held (third principle to maintain researcher well-being).

### Data collection

Quantitative and qualitative data were collected. Quantitative data included participant demographics, proportion recruited out of number approached and participant consent rate (per month). Partway through in-person recruitment researchers began keeping a record of time they each spent on tasks they conducted once an individual who had been approached returned a positive EoI (January – March 2020). When recruitment resumed remotely in September 2020 researchers were asked to continue recording their time but did so till December 2020 only. Thus timesheets were kept at a later stage of the in-person recruitment period when researchers were potentially more familiar with the consent process, but at the early stage of remote recruitment period when researchers were still learning the process.

Participant information sheets informed potential participants that the researcher *might* ask further questions about their experience of the approach and consent process and what they understood the intervention to be, in addition to the study questionnaires and interviews. Participants who had consented to the study were thus given the option to provide additional consent to possibly being asked these questions. However not all who consented to an additional interview were invited to do so; researchers used their judgement on whether to make this offer based on how tired the person with dementia was, or if it was appropriate to do so following the consent and capacity process. To ensure participants’ memory of the process was fresh, questions were asked upon completion of the remote capacity and consent process, before baseline data were collected (if being collected at the same session).

### Data analysis

#### Quantitative analysis

Baseline participant demographic characteristics including gender, age, ethnicity, marital status, living arrangements, carer status, number of currently prescribed medications, diagnostic data including year of dementia diagnosis and type of dementia were summarised descriptively. Means and standard deviations (SD), or median and inter-quartile range (IQR) where appropriate, were used to summarise continuous characteristics, and the number and percentage for categorical characteristics.

We calculated the overall number and percentage of participants recruited out of the number of eligible patients approached. Those consented in person vs. remotely as well as rate of participant consent per month using each method were compared. Mean researcher time to complete consent-related activities was estimated (because researchers were not consistent in recording their time).

#### Qualitative analysis

All qualitative interviews were audio recorded and professionally transcribed. We applied a reflexive thematic analysis to the interviews. We sought to understand participants’ experiences and perceptions of consent meetings through “unstructured and organic” inductive coding and by identifying meaningful patterns across the qualitative data set [35]. The six phases of reflexive thematic analysis [35] guided the analysis process. Two researchers independently read the transcripts and coded the interview transcripts. Initial semantic codes (e.g. participant felt at ease) were developed into latent codes (e.g., relationship building) during a second cycle of coding. The codes were combined to form prominent themes, which were then developed and refined through group discussion and by returning to the data to review initial interpretations***.***

## Results

### Participant baseline characteristics

Among the 56 participants with dementia recruited to the study from 10 GP practices, there was gender balance overall, though more women were recruited during COVID-19 (62% during COVID vs. 32% pre-COVID). The mean age at recruitment was 81.4 years (SD 7.2). Median years since dementia diagnosis was 2.5 years [IQR 1.0;5.0]. Alzheimer's disease was the predominant diagnosis (*n* = 21/39; 54%). Of the 19 participants for whom there was data, 13 were on two or more medications. Most participants were white, married, or in a civil partnership, and lived with their main carer. Overall, about a third of participants (*n* = 18/56; 32%) were able to provide consent, while the rest were consented via consultee. A higher proportion of participants were consented by consultee during COVID-19 (76%) compared to 55% pre-COVID (see Table 2).

**Table 2:** Summary statistics of the PwD by recruitment phase (Pre-COVID or COVID) and in total. Numbers specified in each category if this differs from the total consented

	Pre-COVID			COVID			Overall
N recruited (according to allocation)	TAU [N = 6]	DPACT + TAU [N = 16]	Total [N = 22]	TAU [N = 2]	DPACT + TAU [N = 32]	Total [N = 34]	[N = 56]
Region	[N = 6]	[N = 16]	[N = 22]	[N = 2]	[N = 32]	[N = 34]	[N = 56]
SW, N (%)	5 (83)	15 (94)	20 (91)	2 (100)	12 (38)	14 (41)	34 (61)
Person providing consent	[N = 6]	[N = 16]	[N = 22]	[N = 2]	[N = 32]	[N = 34]	[N = 56]
PwD, N (%)	4 (67)	6 (38)	10 (45)	0 (0)	8 (25)	8 (24)	18 (32)
Gender	[N = 6]	[N = 16]	[N = 22]	[N = 2]	[N = 30]	[N = 32]	[N = 54]
Female, N (%)	2 (33)	5 (31)	7 (32)	1 (50)	19 (63)	20 (62)	27 (50)
Age at baseline	[N = 5]	[N = 16]	[N = 21]	[N = 2]	[N = 30]	[N = 32]	[N = 53]
Median [IQR]	85.4 [80.2;89.3]	78.9 [73.8;86.1]	80.3 [75.1;88.4]	83.8 [82.1;85.5]	83.1 [76.3;86.4]	83.1 [76.9;86.5]	81.5 [76.0;86.8]
Mean (SD)							81.4 (7.2)
Years since diagnosis	[N = 1]	[N = 13]	[N = 14]	[N = 0]	[N = 22]	[N = 22]	[N = 36]
Median [IQR]	1.0 [1.0;1.0]	2.0 [1.0;3.0]	2.0 [1.0;3.0]		3.5 [1.2;6.0]	3.5 [1.2;6.0]	2.5 [1.0;5.0]
Mean (SD)					3.9 (2.7)	3.9 (2.7)	3.3 (2.5)
ACE-III total score	[N = 6]	[N = 13]	[N = 19]				[N = 19]
Median [IQR]	61.5 [55.0;64.2]	70.0 [56.0;72.0]	63.0 [54.5;71.0]				63.0 [54.5;71.0]
Mean (SD)	57.0 (11.7)	64.7 (12.0)	62.3 (12.2)				62.3 (12.2)
TICS-m total score				[N = 2]	[N = 26]	[N = 28]	[N = 28]
Median [IQR]				10.0 [9.5;10.5]	19.0 [15.2;22.8]	18.0 [11.0;22.2]	18.0 [11.0;22.2]
Mean (SD)				10.0 (1.4)	17.6 (7.2)	17.0 (7.2)	17.0 (7.2)
Type of diagnosis	[N = 1]	[N = 13]	[N = 14]	[N = 0]	[N = 25]	[N = 25]	[N = 39]
Alzheimer's, N (%)	1 (100)	9 (69)	10 (71)	0 (0)	11 (44)	11 (44)	21 (54)
Vascular, N (%)	0 (0)	4 (31)	4 (29)	0 (0)	2 (8)	2 (8)	6 (15)
Mixed, N (%)	0 (0)	0 (0)	0 (0)	0 (0)	3 (12)	3 (12)	3 (8)
Other, N (%)	0 (0)	0 (0)	0 (0)	0 (0)	8 (32)	8 (32)	8 (21)
Unsure, N (%)	0 (0)	0 (0)	0 (0)	0 (0)	1 (4)	1 (4)	1 (3)
Number of prescribed medications	[N = 1]	[N = 1]	[N = 2]	[N = 0]	[N = 17]	[N = 17]	[N = 19]
None, N (%)	0 (0)	1 (100)	1 (50)	0 (0)	0 (0)	0 (0)	1 (5)
One, N (%)	0 (0)	0 (0)	0 (0)	0 (0)	5 (29)	5 (29)	5 (26)
Two or more, N (%)	1 (100)	0 (0)	1 (50)	0 (0)	12 (71)	12 (71)	13 (68)
Ethnicity	[N = 6]	[N = 16]	[N = 22]	[N = 2]	[N = 30]	[N = 32]	[N = 54]
White. N (%)	6 (100)	16 (100)	22 (100)	2 (100)	28 (93)	30 (94)	52 (96)
Asian/Indian, N (%)	0 (0)	0 (0)	0 (0)	0 (0)	1 (3)	1 (3)	1 (2)
Caribbean, N (%)	0 (0)	0 (0)	0 (0)	0 (0)	1 (3)	1 (3)	1 (2)
Participant's relationship status	[N = 6]	[N = 16]	[N = 22]	[N = 2]	[N = 30]	[N = 32]	[N = 54]
Single, N (%)	0 (0)	1 (6)	1 (5)	0 (0)	1 (3)	1 (3)	2 (4)
Married/civil partnership, N (%)	6 (100)	11 (69)	17 (77)	1 (50)	23 (77)	24 (75)	41 (76)
Divorced/civil partnership dissolved, N (%)	0 (0)	0 (0)	0 (0)	1 (50)	1 (3)	2 (6)	2 (4)
Widowed/surviving civil partner, N (%)	0 (0)	4 (25)	4 (18)	0 (0)	5 (17)	5 (16)	9 (17)
Participant's living status	[N = 6]	[N = 16]	[N = 22]	[N = 2]	[N = 30]	[N-32]	[N = 54]
Alone, N (%)	0 (0)	2 (12)	2 (9)	1 (50)	5 (17)	6 (19)	8 (15)
With main carer, N (%)	5 (83)	12 (75)	17 (77)	1 (50)	17 (57)	18 (56)	35 (65)
With main carer and others, N (%)	1 (17)	2 (12)	3 (14)	0 (0)	6 (20)	6 (19)	9 (17)
Other, N (%)	0 (0)	0 (0)	0 (0)	0 (0)	2 (7)	2 (6)	2 (4)

### Participant recruitment

#### Proportion recruited from those approached

The lockdown in March 2020 caused us to pause mid-way through our four-stage approach of patients at the eight GP practices that had been recruited to the study. Those who had already expressed a positive EoI were notified of a pause; permission was sought to resume contact once the trial was un-paused. After the study resumed in September 2020, three of the original GP practices (SW2 [Southwest practice # 2], NW3 [Northwest practice #3], and NW4) continued to experience staff shortages intensified by COVID-19 and were unable to continue with the study. The three SW practices remaining in the study were large in comparison with the two remaining NW practices and had potentially enough people with dementia who had not yet been approached to allow us to reach the SW recruitment target. This was not so for the NW practices, and so two additional NW practices (NW5 and NW6) were recruited in October 2020. Unfortunately, both of these practices withdrew before participant recruitment commenced, citing COVID-19 research and lockdown 2 (November 2020) as contributing factors. A further two NW practices (NW7 and NW8) were recruited in December 2020; both were allocated to the intervention group to maximise our learning for Phase 1 Feasibility. Researchers remained blind to these allocations until at least post-baseline data collection; unblinding often occurred at follow-up based on participant responses.

A total of 421 potentially eligible patients were approached (*n* = 223 pre-COVID-19, *n* = 198 during COVID). 58 patients were consented, but two were subsequently excluded after completing the TICS-m as their score was greater than 29 i.e. not meeting the inclusion criterion. Thus 56 patients with dementia (13.3% of those approached) entered the study, 50.9% of the overall Phase 1 recruitment target (56/110). When SW1 and NW1 (non-randomised practices) were excluded, the number recruited was 39 out of 368 approached (10.6%). All recruited patients had carers, who consented to participate alongside their spouse/parent/friend. One participant withdrew post-TICS-m but before baseline measures were collected. Individual practice-level recruited/approached rates varied from 6 to 33% (final column, Table 3).

**Table 3:** Number of participants recruited at each GP practice by period (pre-COVID and COVID)

	Pre-COVID (Sept 2019-March 2020) In-person			During COVID (Sept 2020-March 2021) Remote				Overall % recruited/approached per practice
	Approached (n)	Consented (n)	Recruited/approached (%)	Consented *during* COVID-19 but approached *before* COVID-19 (n)	Approached during COVID-19 (n)	Consented and approached during COVID-19 (n)	recruited/approached (%)	
SW1	29	12	41	-	19	4	21	33
SW2	48	3	6	1	-	-	-	8
SW3	26	-	-	2	-	-	-	10
	-	-	-	-	47	5	11	
SW4	25	5	20	2	-	-	-	19
	-	-	-	-	12	0	0	
NW1	5	1	20	-	-	-	-	20
NW2	17	1	6	-	-	-	-	6
NW3	18	-	-	4	-	-	-	22
NW4	55	-	-	6	-	-	-	11
NW7	-	-	-	-	60	4	7	7
NW8	-	-	-	-	60	6	10	10
*Total*	*223*	*22*	*9.9%*	*15*	*198*	*19*	*9.6%*	*13.3%*

*SW* Southwest, *NW* Northwest, *SW1* Southwest practice # 1, etc.

**Fig. 1:**
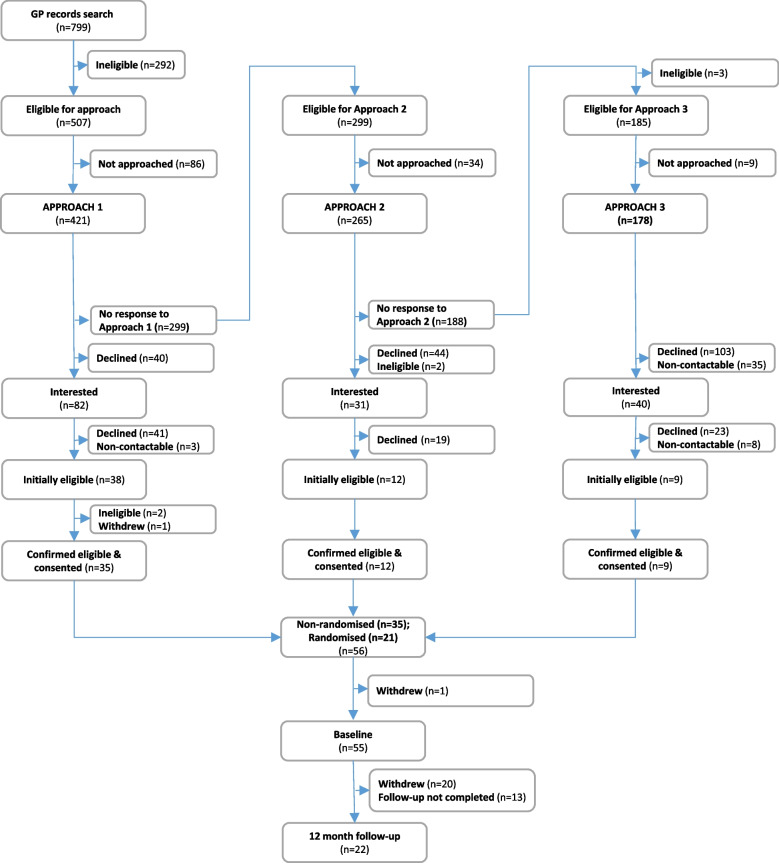
CONSORT diagram for overall recruitment (in-person and remote combined)

As described elsewhere [10] and summarised in Appendix A, participants were approached using a multi-stage strategy where people who had not responded to an approach received a further approach (up to a maximum of four approaches before the pandemic and three during the pandemic). The total number of consented participants are summarized in the CONSORT below (Fig. 1). The proportion recruited from total approached at Approaches 1, 2 and 3 was 8.3%, 4.5% and 5.1% respectively, or 42.7%, 38.7% and 22.5% of those interested (i.e. returned a positive EoI) respectively. Approach 4 (welfare check via a home visit) was implemented at one practice (SW1) before being suspended due to government guidelines restricting in-person contact. Only three potential participants received Approach 4 (all from SW1), out of which one was consented. For reporting purposes this individual has been added to the Approach 3 recruitment total shown in tables and the CONSORT (Fig. 1).

Before COVID-19 the total number consented in-person from all practices (non-randomised and randomised combined) was 22 (out of 223 approached, or 9.9%). During the COVID-19 period, 34 people with dementia were consented remotely. Fifteen of the 34 remotely consented participants were part of a group of patients approached before the pandemic; following all trials being paused at the start of the national lockdown these patients were informed of the pause and placed “on hold” temporarily (Table 3, shaded column). These individuals were at different stages in the approach process when the study was paused: some had returned a positive EoI and were waiting for more information; some had returned a positive EoI, had already received information and were considering whether to go forward; others had agreed to go forward and were pending a consent meeting (or a date for a consent meeting). After the study was un-paused, these individuals who had provided permission to do so were re-approached. Those still interested to proceed and take part underwent consent using our new remote process. Excluding these 15 consented participants, there were 19 (out of 198 approached; 9.6.%) who were approached *and* consented during COVID-19 (penultimate column, Table 3).

We also examined all remotely consented participants’ (*n* = 34) preferred method for remote communication. Overall, the telephone was the preferred option for 21 (61.8%) of the 34 participants. The telephone was more favoured in the SW: 12 of the 14 SW participants (85.7%) chose the telephone compared to 9 of the 20 (45%) of NW participants.

#### Rate of participants consented per month

In-person recruitment occurred between 1st September 2019 and 4th March 2020; recruitment resumed remotely (after an imposed pause due to COVID-19) on 1st September 2020 and stopped 4th March 2021. During the combined recruitment period of 52 weeks and 5 days, mean participant consent rate was 4.6 participants per month. In-person consent progressed at a mean rate of 3.6 participants per month. In total we were able to approach or re-approach and remotely consent 34 participants over a similar recruitment period length of approximately 26 weeks, at a mean rate of 5.6 participants per month (Table 4). As above in Table 3, Table 5 separates those participants (*n* = 15; see Table 3, (%)Consented during COVID-19 but approached before COVID-19 (n)) who had been approached before the pandemic lockdown but had to be put on hold, had agreed to be re-contacted once the trial was un-paused and who had agreed to take part. These participants who agreed to take part were consented remotely during COVID-19, at a mean rate of 2.6 participants a month. The mean participant consent rate of those who were approached and consented during COVID-19 (*n* = 19) was 3 per month (Table 5).

**Table 4:** Participant consent rates by period (pre-COVID-19 and COVID-19)

Recruitment period	Duration	Number of people with dementia (PwD) consented (n)	Participant consent rate per week	Participant consent Rate Per Month (1 month = 4.3 weeks)
Pre-COVID-19 (in-person consent)	26 weeks and 3 days	22	0.8	3.6
COVID-19 (remote consent)	26 weeks and 2 days	34	1.3	5.6
Total active	52 weeks and 5 days	56	1.1	4.6

**Table 5:** Remote participant consent rates by those reapproached vs. freshly approached during COVID-19

Recruitment period (remote only)	Duration	Number of people with dementia (PwD) consented (n)	Participant consent rate per week	Participant consent Rate Per Month (1 month = 4.3 weeks)
Approached *before* COVID-19/consented during COVID-19	26 weeks and 2 days	15	0.6	2.6
Approached *and* consented during COVID-19		19	0.7	3.0

#### Time required to gain informed consent

A returned positive EoI signalled permission to be contacted by a researcher. Five types of activities followed this point: (i) pre-consent activities, e.g. preparatory telephone calls made to the participant (during which researchers might address any participant questions), preparation for the session (including materials), updating records; (ii) consent/baseline; (iii) post-consent activities (storing consent, updating records, researcher reflections, relevant correspondence (GP, participant); (iv) other miscellaneous activities, e.g. any additional or unscheduled tasks, such as ad-hoc participant phone calls to researchers and (v) travel. Table 6 shows the mean time per participant for each of these consent-related activities (total researcher time summed across all researchers for a given activity over the reporting period, divided by number of participants recruited during that period). Because timesheets were kept for only part of each recruitment period, the mean time was calculated based on number of participants with dementia recruited *only* during those months (*n* = 12 for in-person; *n* = 18 for remote).

**Table 6:** Average time per participant for consent activities in in-person and remote settings

Activity	In-person Mean time per participant (mins)	Remote Mean time per participant (mins)
Pre-consent activities	69	384
Consent/baseline*	188	201
Post-consent activities	40	137
Other	0	109
Travel	253	N/A
Mean time spent per participant for all activities (researcher-hours)	550 (9^ hours)	831 (14^ hours)

^*^(i) During in-person recruitment, collection of baseline measures was conducted immediately after consent for 19/22 (85%) participants with dementia, usually at the request of the participants although researchers also used their judgement about whether a participant was too fatigued to continue. As a result researchers recorded the combined time for consent and baseline. When recruitment switched to remote methods during the pandemic participants were more likely to request for baseline data to be collected at a separate time (22/34 or 65% participants with dementia). For fair comparison between in-person and remote methods, time for both consent and baseline are combined

(ii) Time taken to complete interview questions on the consent/capacity process is not included

^Rounded up from 8.9 h and 13.9 h respectively

Timesheets indicated that overall, more hours per participant on average were required for remote consent (14 researcher-hours) compared to in-person consent (9 researcher-hours). Researchers travelled a mean of 253 minutes per participant to attend and return from in-person meetings. Although no travel took place during the pandemic (remote recruitment) the length of time spent on pre-consent, post-consent and other tasks increased by about six times (Table 6). However, mean researcher-hours spent on the actual consent and baseline meetings in-person vs. remotely were not much different (mean 188 vs. 201 min respectively). During in-person recruitment two researchers attended the consent/baseline meeting, compared to remote recruitment where a single researcher conducted the meeting. This difference means that while researcher-hours were comparable across in-person and remote recruitment, the participant’s experience of the meeting duration differed. That is, in-person meetings on average would have lasted approximately 90 min while remote meetings on average would have lasted more than double this time (201 min).

### Qualitative results

**Fig. 2:**
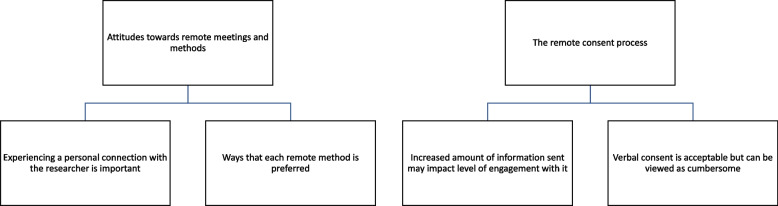
Overview of themes and sub-themes

For the purpose of this paper we focused on interviews relating to activities from the point the researcher contacted the potential participant (with permission) to the consent and capacity meeting during COVID-19 (*n* = 13). All interviewees were consented remotely. However two of these 13 interviewees were part of the 15 participants whose pre-COVID 19 approach had been interrupted because of the lockdown and who had been reapproached when the study was un-paused and had agreed to take part and consented remotely. Interviews were brief, lasting from 4–21 min. Two themes, each with sub-themes, were identified (Fig. 2).

#### Theme 1: attitudes towards remote meetings and methods

The first theme was related to interviewees’ attitudes towards the communication mode for remote consent meetings.

##### Sub-theme 1.1: experiencing a personal connection with the researcher is important

Interviews suggest that what is valued by participants is feeling a personal connection with the researcher. Implied in their comments is the perception that in-person meetings are the best way to achieve this because it allows them to “see a face” or “have human contact” (e.g. 10301). This may explain why in-person meetings was the stated preference for many of the interviewees, had they been possible during the lockdowns. This is further supported by the admission among interviewees that in the absence of an in-person meeting, video calls would be the “next best thing” (e.g. 10204). We return to this point in the Discussion.

The mode of communication became less important *if* the researcher was able to inspire that feeling of a personal connection over the telephone. For example, empathetic and friendly communication styles positively influenced participants’ view of remote meetings and encouraged a sense of comfort and openness with the researcher.

##### Sub-theme 1.2: ways that each remote method is preferred

Although video calls were thought to be better than telephone calls because it allowed potential participants to “see” the researcher’s face, it could also on the other hand make it more difficult to focus. For example, one carer (20402) described how their family member with dementia found that the reduced stimuli associated with a telephone call helped them to concentrate better on what the researcher was saying and made it easier for them to respond. 

Additionally, remote meetings – whether by telephone or video call – were felt to be easier to terminate compared to in-person meetings in the event of an unexpected development, such as the person with dementia becoming anxious (e.g. 20801).

#### Theme 2: views on the remote consent process

The second theme was related to the consent process itself and how interviewees viewed the steps we put into place (information sent, verbal consent) to facilitate the remote consent meeting.

##### Sub-theme 2.1: increased amount of information may impact level of engagement with it

As described above, during COVID-19 participants received a copy of baseline questionnaires and any supporting documentation *in addition* to study information prior to a scheduled remote meeting. Although there was understanding as to why this was needed, many interviewees agreed that there was quite “a lot of it” (10304). In terms of study information (including the PIS), views ranged from it being repetitive, heavy or dull (e.g. 20301).

Sending the study information, supporting documents and baseline questionnaires together may have led some to feel that they needed to look at everything (“you realise that everything has to be covered”, 20301) and as a result to skim the material instead (104-m). This may have impacted on the engagement with materials and quality of understanding about the study for some, especially regarding the dementia support worker's role (e.g. 10h116, 10304).

##### Sub-theme 2.2: verbal consent is acceptable but can be viewed as cumbersome

Consent was obtained verbally during remote recruitment. Responses like 10116’s illustrate that while verbal consent was “fine” (none of the interviewees expressed a problem with it), it could also be seen as time-consuming compared to reading and initialising each statement themselves.

## Discussion

The aims of this paper were to (i) describe how our in-person consent and capacity judgement process for people with dementia was adapted for remote working during the pandemic lockdowns and restrictions; (ii) describe the impact this had on our recruitment performance and (iii) describe how a subset of participants felt about these adapted processes. The adaptations we made are described above (aim i). The impact of these adaptations on recruitment (aim ii) and participant views on them (aim iii) are discussed here, following a brief discussion of our overall recruitment.

### Overall recruitment performance

Recruiting individuals with dementia from primary care (i.e. who are not in-patients or being seen regularly by secondary care) is inherently challenging [1, 6]. Although one study recruiting from primary care to a cluster RCT aiming to improve dementia care achieved an overall recruitment rate of 54% [6], rates as low as 3.1% of those approached have been reported [36]. The latter, a non-randomised feasibility study for a trial on withdrawing antihypertensive medication, recruited 30 people with dementia (some of whom lacked capacity to consent). In our feasibility study we consented a total 56 participants with dementia (13.3% of those approached, in-person and remote recruitment combined), which was on the lower side of this range.

### How did remote consent compare with in-person consent?

The pandemic introduced additional complexities but also created the opportunity to test remote recruitment strategies in primary care.

#### Proportion recruited out of those approached

Switching to remote consent during the pandemic did not adversely affect the number of participants we were able to recruit (Table 3). We compared the proportion of participants consented out of the total approached and found similar participant consent rates during the recruitment period prior to COVID-19 (in person) and the period during COVID-19 (remote), i.e. 9.9% vs. 9.6% respectively (this does not include the 15 participants who were re-approached and consented remotely). Both recruitment periods were of similar length and occurred during the same timeframe (September to March). The latter is noteworthy, given that there is known seasonal variability in recruitment [2, 3].

#### Rate of consent per month

The consent and capacity judgement component of recruitment was the most challenging to adapt to a remote process, given the cognitive and sensory challenges often associated with dementia. Our combined (in-person + remote) mean rate of consent of 4.6 (Tables 4 and 5) is comparable to other studies on psycho-social interventions for dementia, which have reported a range of 2–4 participants per month [2, 3]. We believe that the mean number of participants per month consented remotely (5.6 participants per month) surpassed that done in person (3.6 participants per month) partly because travel time was eliminated. Not having to travel to participant homes (rarely under an hour each way) made it possible to consent more than one participant/dyad in a day, although this did depend on the participant’s level of need – we did not rush participants if they needed time. Another potential reason for the higher rate of consent using remote methods was that it was easier to secure appointments for telephone or video meetings (which could be split over more than one session) with potential participants, compared to an in-person meeting where the dyad would need to ensure they were home or could meet at their GP surgery for the length of the consent meeting (pre-pandemic this was usually more than an hour). This, combined with people being at home during the lockdowns, increased the likelihood of more regular (weekly) scheduling of remote consent meetings. With two researchers often involved in the consent visit prior to COVID-19, this also meant fewer potential participant meetings could be scheduled each month. During remote consent, where a single researcher attended to the consent/baseline via telephone or video, more than one individual or dyad could be scheduled simultaneously.

#### Average time to consent a participant

Researchers kept timesheets for consent-related tasks from the point of a positive EoI to gaining informed consent. Although we did not have complete records over the two recruitment periods, we were able to estimate the time required to consent in person vs. remotely. The mean time required per participant to conduct all consent-related activities was higher remotely (14 researcher-hours) than in person (9 researcher-hours). Researcher-hours for the consent and baseline meeting (see Table 6 note for why the timing was combined) was comparable in person and remotely (188 vs. 201 mins respectively). However it should be noted that while per-participant researcher-hours were comparable, participant-experienced meeting duration was not. In-person researcher-hours were summed across two researchers (two researchers attended the consent/baseline meeting) so duration of in-person meetings was half of the researcher-hours reported. Remote meetings were conducted by one researcher and so reported researcher-hours also reflect the length of the meeting experienced by the participant. Almost all in-person consent and baseline meetings were completed in one sitting compared to only about one third of remote meetings (where consent was completed in one sitting but baseline meetings at subsequent meetings). On average, almost 4 h per participant was spent on travel, which accounted for just over 40% of total mean time required to consent a participant in person. The increase in total mean time required to complete all remote consent-related activities was due to increased preparation, responding to enquiries before the consent meeting and administrative tasks (approximate average of 10.5 man-hours per participant out of 14, or 75%). When recruitment was in person, pre-consent activities (i.e. before the study involvement meeting) comprised a phone-call to agree a time to visit, the printing and posting of the patient information sheet and consent form as well as the printing of other materials to be brought to the meeting. Often printing was done in advance and stored at the office. During the pandemic researchers were at home and needed to print and post everything on their own. Prior to COVID-19, researchers may have had to answer queries over the telephone on occasion, but questions tended to be asked during the consent meeting. While pre-consent telephone calls to agree visit time still happened during the pandemic (remote recruitment), researchers also reported receiving many more telephone calls from potential participants before a consent meeting, usually with questions about the upcoming meeting (see “other”, Table 6). We speculate that potential participants may have been more anxious about being consented remotely or may have craved some kind of contact due to a general sense of loneliness and isolation – exacerbated by COVID-19 for many [37]. Researchers also used some time preparing for the telephone or video call, e.g. calling potential participants to perform technical checks or demonstrate/answer any questions about this. Post-consent activities took longer as well; for example, prior to COVID-19 physical consent forms were scanned before both scanned and physical forms were stored securely. During COVID-19 researchers also had to process audio recordings of verbal consent following the consent call, on top of securely processing paperwork.

As reported above, researcher timesheets were available for only Jan-Mar 2020 pre-pandemic (i.e. the latter half of the in-person recruitment period) and during the pandemic records were kept from Sept-Dec 2020 (the first half of the remote recruitment period). It is possible that the longer hours required for remote recruitment reflects (in part) researchers getting used to the unfamiliar remote process. Another potential factor contributing to increased researcher-hours during remote recruitment relates to researcher debriefing and reflections. During in-person visits, researchers travelled to and from participant homes in pairs. Debriefings sometimes happened during the return journey, whereas during remote recruitment these debriefings would take place via a separate call with other researchers (thus adding to time spent on post-consent activities).

### Impact of remote working on researchers

Our researchers experienced challenges related to remote interactions such as fatigue, also reported by others [38]. Judging capacity was another challenge. Remote working removed the possibility of researchers working in pairs as we did pre-COVID-19 [10]. Dual working allowed researchers to consult with each other when gauging capacity and increased confidence with capacity judgements. During remote recruitment, capacity judgement was made by a sole researcher; less experienced researchers felt more pressure as a result. We have shared some ways that we addressed these challenges elsewhere [39], which included debriefing sessions for researchers as well as ongoing support and supervision.

### How did participants feel about being consented remotely?

The advantages of including qualitative research into primary care RCTs to explore recruitment issues have been highlighted [40]. Qualitative interviews about the remote consent process (that utilised a combination of post, email, telephone or video calls) revealed that it was generally acceptable to participants. Person-centred (tailored) communication practices were key to promoting a connectedness to the researcher, which interviews suggest were important to participants. We believe that the stated preference for in-person meetings or at least a video call reflected interviewees’ belief that connectedness is achieved when a face can be seen. However, researchers were able to demonstrate that rapport can also be achieved over the telephone (but may take longer) by adopting an empathetic and friendly manner. Having one researcher assigned to a participant was important in building a relationship, especially during remote working. The increased number of telephone calls with participants that we observed during this time increased time required to complete a consent process but were beneficial to helping participants and the researcher feel more comfortable with each other as well.

While interviewees created the impression that video calls were closer to in-person meetings because participants could see the researcher, more than half (62% of 13) of remotely consented participants were consented via telephone. This lack of congruence may reflect a bias in the sample interviewed. Interviews suggest that not everyone was comfortable with video calls – visual stimuli could be distracting for some people with dementia – nor equipped for them, especially in the SW (which included GP practices in rural areas) where telephone was the predominant choice.

One perceived advantage of remote meetings (telephone or video) among interviewees was that it made it easier to cut a call short if they needed to, e.g. if the person with dementia began to feel too anxious or tired. Researchers in the SW had to travel fairly long distances when consent meetings were in person and we believe these views suggest this may have made participants less comfortable about asking the researcher to return another time.

Interviews with a subset of consented participants suggest that one area with room for improvement was around the amount and clarity of the information that was sent to potential participants prior to a remote consent meeting. Although this view was obtained from interviews about the remote process, these lessons could apply to in-person consent meetings as well. As described above, during remote recruitment, potential participants received the PIS, consent form, baseline questionnaire booklets and supporting documentation *prior* to consent calls, increasing information that potential participants received compared to in-person recruitment. Some individuals found the information repetitive and overwhelming in amount despite our best efforts to reduce material. Lockdown conditions at the time meant that researchers had to print materials from their home and go out to post these materials prior to a consent meeting. As there were restrictions on leaving the home, all materials were sent in one package. Potential participants coped by not reading the information “word-for-word”, aiming instead to understand the “gist”. This raises the question of whether sending all the information that is usually required for a typical consent actually undermines its purpose of the individual making an informed decision. It also potentially explains gaps in some consented participants’ understanding of the intervention (the DSW role), although it is also possible that because we were still in the intervention development phase researchers themselves were less able to provide clarity.

These learning points informed our recruitment plans for Phase 2 of D-PACT. For example, only essential information was included in the PIS, with additional details provided separately in a “Further Information Sheet”. Clearer communication about the DSW’s role was provided. Language was made even more accessible and materials more visually friendly. An easy-read list of questions replaced the original questionnaire booklet (questionnaires in their original form). Information was also sent in stages rather than one go. Adjustments made for Phase 2 demonstrate a responsive approach to enhance participant understanding and engagement.

In summary, our experience indicates that remote consent of people with dementia to a trial, though not without challenges, can be person-centred and efficient. Importantly, our findings show that a fully remote consent process did not compromise recruitment rates. Our study suggests that when circumstances do not allow in-person contact, remote methods are a good (enough) and efficient alternative, with the longer mean time required per participant for consent counter-balanced by savings in travel time and more consent meetings scheduled in a week. This learning prompted a hybrid approach in Phase 2 (Evaluation) of our study, offering participants flexibility and choice and allowing mode of communication to be dictated by participants rather than a rigid protocol.

### Limitations

Our findings stem from recruitment in only two settings with a modest total number of recruits and partial data for tracked researcher time, potentially limiting generalisability. Additionally, interviews were with remotely recruited participants only; there was also a potential selection bias in our sample because researchers were instructed to use their discretion (e.g. checking the level of fatigue in the participant) on whether to ask the participant additional questions after the consent process was completed. While our participants did have varied capacity levels, other characteristics indicate a relatively homogenous group of participants who were primarily white British, in married or civil partnerships, residing with their main carer. Demographic data for those declining participation are absent, preventing insights into recruitment processes in more diverse or lower-income communities, an aspect explored in Phase 2 (Evaluation) of D-PACT.

## Conclusions

Limitations notwithstanding, we conclude that remotely judging the capacity of and consenting people with dementia – including those who lack capacity to consent – is not only possible but results in comparable rates to in-person consent. Moreover, primary care-based recruitment is viable, yielding over 10% of approached individuals, albeit requiring time. Researchers should prioritise flexibility when recruiting people with dementia; our findings suggest that it is possible to provide choice (remote or in-person methods) without compromising recruitment performance. Our study contributes to published recruitment strategies for trials based in primary care, emphasising that remote consent of participants with dementia (including those who lack capacity to consent) can be as productive as in-person consent. Understanding the time commitment required to consent people with dementia enables researchers to set realistic targets and align designs with their aims and population. Funders should recognize these timeframes (and therefore financial requirements [41]) for recruiting to dementia trials based in primary care in their decision-making processes.

## Data Availability

No datasets were generated or analysed during the current study.

## Appendix

### Comparison of pre-pandemic identification and approach components of the recruitment strategy

**Table:**

Recruitment component	In-person (pre-COVID-19)	Remote (during COVID-19)
Identification List of patients with a diagnosis of dementia screened for eligibility	Research nurse did this at the practice	Research nurse did this remotely
Approach Four-stage approach where: - Patients returning positive expressions of interest were contacted by the researcher and if willing went on to attend a consent meeting (study involvement meeting) - Negative expressions of interest were noted and not contacted again - Non-responders went on to receive the next approach	Approach 1: letter and study information to all eligible patients Approach 2: for Approach 1 non-responders. Letter, invitation to attend a study information meeting at the GP surgery OR over the telephone Approach 3: for Approach 2 non-responders. Welfare check over telephone and if appropriate an offer to learn about the study Approach 4: for Approach 3 non-responders. Welfare check at home and if appropriate an offer to learn about the study	Approach 1: letter and study information to all eligible patients Approach 2: for Approach 1 non-responders. Letter, invitation to call researchers at designated times to find out more Approach 3: for Approach 2 non-responders. Welfare check over telephone and if appropriate an offer to learn about the study Approach 4: Did not happen

The D-PACT recruitment strategy comprised three components: identification, approach and consent/capacity judgement, see also Griffiths et al., 2022. Patients who agreed to a consent meeting at any point in the approach underwent the consent and capacity judgement process. This is described in the main text.

## Abbreviations

ACE-III: Addenbrooke’s Cognitive Examination III
cRCT: Cluster randomised controlled trial
CMHT: Community and mental health teams
CONSORT: CONsolidated Standards Of Reporting Trials
CRN: Clinical Research Network
D-PACT: Dementia PersonAlised Care Team
DSW: Dementia support worker
GP: General practice
IMD: Index of Multiple Deprivation
IQR: Interquartile range
NIHR: National Institute for Health and Care Research
NW: Northwest
QOF: Quality and Outcomes Framework
SNOMED-CT: Systematised Nomenclature of Medicine Clinical Terms
SW: Southwest
TAU: Treatment as usual
TICS-m: Modified Telephone Interview of Cognitive Status
UK: United Kingdom
